# An Efficient *T*_1_ Contrast Agent for Labeling and Tracking Human Embryonic Stem Cells on MRI

**DOI:** 10.1155/2019/3475786

**Published:** 2019-06-16

**Authors:** Inga E. Haedicke, Sadi Loai, Hai-Ling Margaret Cheng

**Affiliations:** ^1^Institute of Biomaterials & Biomedical Engineering, University of Toronto, 164 College Street, RS407, Toronto, ON, Canada M5S 3G9; ^2^Ted Rogers Centre for Heart Research, Translational Biology & Engineering Program, Toronto, Canada; ^3^The Edward S. Rogers Sr. Department of Electrical and Computer Engineering, University of Toronto, Canada; ^4^Heart & Stroke/Richard Lewar Centre of Excellence for Cardiovascular Research, Toronto, Canada; ^5^Ontario Institute for Regenerative Medicine, Toronto, Canada

## Abstract

Noninvasive cell tracking in vivo has the potential to advance stem cell-based therapies into the clinic. Magnetic resonance imaging (MRI) provides an excellent image-guidance platform; however, existing MR cell labeling agents are fraught with limited specificity. To address this unmet need, we developed a highly efficient manganese porphyrin contrast agent, MnEtP, using a two-step synthesis. In vitro MRI at 3 Tesla on human embryonic stem cells (hESCs) demonstrated high labeling efficiency at a very low dose of 10 *µ*M MnEtP, resulting in a four-fold lower *T*_1_ relaxation time. This extraordinarily low dose is ideal for labeling large cell numbers required for large animals and humans. Cell viability and differentiation capacity were unaffected. Cellular manganese quantification corroborated MRI findings, and the agent localized primarily on the cell membrane. In vivo MRI of transplanted hESCs in a rat demonstrated excellent sensitivity and specificity of MnEtP for noninvasive stem cell tracking.

## 1. Introduction

Cell transplantation therapy based on human embryonic stem cells (hESCs) has great potential in treating complex medical conditions such as cardiovascular disease, autoimmune disease, cancer, and neurological disorders [1, 2] as they are pluripotent and have unlimited proliferation potential [3, 4]. However, while these cell-based therapies show considerable promise, there remain a number of critical barriers related to cell delivery that must be overcome. Currently, we rely on histology to confirm engraftment, a procedure useful strictly in animal studies but not in the clinical setting. The ability to noninvasively visualize the therapeutic cells in vivo would help verify their initial distribution, their continued survival, engraftment, and correlation with improved organ function. Amongst the imaging modalities available for noninvasive cell tracking, magnetic resonance imaging (MRI) is the only nonirradiative modality that allows deep-tissue imaging in addition to providing superb soft tissue contrast, submillimeter spatial resolution, and both anatomical as well as physiological information [5, 6]. Cellular MRI has most commonly been performed with superparamagnetic iron oxides (SPIOs) for its high sensitivity [5]. Unfortunately, this dark-contrast approach suffers from obliteration of signal in surrounding anatomy and nonspecificity in regard to the source of dark signal, be they microbleeds, air/tissue interface, or released SPIO internalized by macrophages [7, 8].

To address these limitations of SPIOs, bright-contrast cell tracking using paramagnetic *T*_1_ agents has also been explored [9]. Recently, a novel cell-permeable manganese porphyrin-based contrast agent, MnAMP, was reported for highly efficient *T*_1_ labeling of mouse embryonic stem cells and human cancer cells [10, 11]. However, the synthetic complexity of this agent makes it difficult to scale up for larger animal models that are required for clinical translation. A second-generation agent, MnPNH2, with a scalable synthesis was developed, and although this agent displayed excellent biocompatibility, its cell permeability was limited relative to MnAMP and a five-fold higher labeling concentration was required to provide sufficient relaxivity for in vivo cell tracking [12]. To maintain cell permeability and a simplified synthesis, we have developed a third-generation manganese porphyrin with a scalable two-step synthesis [13], similar to MnPNH2 but with higher cell uptake and a reduction in *T*_1_ relaxation times comparable to MnAMP but at ten-fold lower labeling concentration. This new agent, named MnEtP, was previously used to treat oxidative stress in a preclinical model of colitis [14]. Here, we report its first demonstration as a cell labeling agent for MRI.

In this work, we investigated the potential of labeling hESCs with MnEtP and sensitively tracking the labeled stem cells in vivo. In keeping with our overall goal of designing safe cellular contrast agents, tests were performed to minimize dose and confirm the absence of negative effects on cell viability, proliferation, and differentiation capacity. Our results showed that the proposed bright-contrast *T*_1_ agent is safe, provides efficient signal enhancement in vivo, and can be scaled up to enable labeling of large cell numbers required for preclinical animal models and eventual translation to human patients.

## 2. Materials and Methods

### 2.1. Human Embryonic Stem Cell Line and Cell Culture

Human ESCs (line ESI-017, ESI Bio, SKU: ES-700) were cultured under sterile conditions on tissue culture flasks and plates coated with Corning™ Matrigel™ Membrane Matrix (Fisher Scientific Cat. No. 08-774-552) and kept in an incubator at 37°C and 5% CO_2_. Cells were maintained in mTeSR™1 (STEMCELL Technologies Cat. No. 85850) and passaged using an enzyme-free, Gentle Cell Dissociation Reagent (STEMCELL Technologies Cat. No 07174) and a mechanical cell scraper for detachment, to prevent differentiation and allow cells to remain in small colonies. Cells were grown in T-75 tissue culture flasks for labeling, and media was changed every 24 h. Cell morphology was monitored every 24 h by light microscopy.

### 2.2. Cell Labeling Studies

To ensure sterility, cell labeling was performed in a biological safety cabinet. MnEtP was dissolved in sterile dimethyl sulfoxide (DMSO) to make 10 mM and 1 mM stock solutions. The stock solutions were sterile filtered into autoclaved vials. The stock solutions were stored in the fridge in between experiments. The contrast agent was added to a specific volume of mTeSR™1 media, mixed and then added to the hESC for a specific labeling period. The concentration of DMSO in media was ≤0.5% for all labeling studies. After labeling, the media was aspirated. The cells were rinsed with room temperature Dulbecco's phosphate-buffered saline (D-PBS) three times, 1 min/rinse, to remove residual agent not taken up by cells. Fresh media was then added to the cells to allow them to continue growing for viability testing, or the cells were detached and pelleted for MRI and quantification or for cell fractionation and quantification.

The colonies were detached by use of a gentle cell dissociation reagent followed by cell scraping and collection. Cells were centrifuged at 300 × *g* for 5 min, and the supernatant was aspirated. The cells were resuspended with 1 mL D-PBS, and 0.9 mL of the cell suspension was transferred to 115 × 5 mm Wintrobe sedimentation tubes (Kimble Chase, Vineland, NJ). The cells were pelleted at 300 × *g* for 5 min, placed in falcon tubes, and sealed and transported on ice to the MR scanner. Optimization of the cell labeling procedure was done by testing a range of agent concentrations and labeling times, from 2 *μ*M–10 *μ*M labeling for 24 h and 10 *μ*M–40 *μ*M labeling for 30 min.

### 2.3. In Vitro MRI

After transportation to the MR scanner, the cell pellet-containing tubes were removed from ice and placed in a custom-made ULTEM™ resin holder. At different intervals after cell labeling (0–2 hours, 24 hours, 48 hours, and 72 hours), imaging was done on a clinical scanner (Achieva 3.0 T TX, Philips Medical Systems) using a 32-channel head coil. High resolution *T*_1_-weighted images were acquired using a two-dimensional (2D) spin-echo sequence: repetition time (TR) = 100 ms, echo time (TE) = 14.1 ms, 60 mm field-of-view (FOV), 2.5 mm slice thickness, 0.5 mm × 0.5 mm in-plane resolution, and number of signal averages (NSA) = 8. *T*_1_ mapping was performed using inversion recovery turbo spin echo: TR = 3000 ms, TE = 18.5 ms, 60 mm FOV, 2.5 mm slices, 0.5 × 0.5 mm in-plane resolution, and inversion time (TI) = (50, 100, 250, 500, 750, 1000, 1250, 1500, 2000, and 2500) ms. After image acquisition, the data were analyzed on a 2.5 mm deep cylindrical volume within each cell pellet. *T*_1_ values were calculated on a pixel-by-pixel using in-house software developed in Matlab (v. 8.1). Quantitative *T*_2_ relaxation times were measured using a 2D multiecho spin-echo sequence: TR = 2000 ms, 32 echoes with minimum TE = 7.6 ms and 7.6 ms echo spacing, 60 mm FOV, 2.5 mm slice thickness, and 0.5 mm × 0.5 mm in-plane resolution.

### 2.4. Subcellular Distribution of MnEtP

Labeled cells were washed as described above and pelleted at 300 × *g* for 5 min. The cells were resuspended in 1 mL D-PBS with 0.01% saponin (Alfa Aesar Cat. No. A18820). After 30 min at room temperature, the cells were centrifuged at 1000 × *g* for 5 min. The supernatant was collected as the cytosolic fraction. To the remaining pellet was added 500 *μ*L D-PBS, followed by 50 strokes on a Dounce homogenizer. After centrifugation at 15,000 × *g* for 15 min, the supernatant was collected as the nuclear fraction and the remainder of the pellet was collected as the membrane fraction. The cytosolic and nuclear fractions were passed through a 0.22 *μ*m PES syringe filter and analyzed by reversed-phased high-performance liquid chromatography (RP-HPLC); for program details, see supporting information. The membrane fractions were digested with 300 *μ*L ultrapure HNO_3_ for 7 h at 40°C. The solutions were diluted to 2% HNO_3_, filtered over 0.22 *μ*m PES syringe filter, and analyzed by inductively coupled plasma atomic emission spectroscopy (ICP-AES) as described above.

### 2.5. Microscopy

Bright field microscopy was used to visually assess cell growth and development throughout each experiment to ensure consistency and viability. Human ESCs were assessed every 24 h as well as before/after each labeling period. Confluency was monitored daily to ensure that the cells never exceeded 80% confluency. As expected, the hESCs maintained a spherical morphology and grew in tightly packed colonies. Proliferation and development were closely monitored after labeling periods to confirm that the agent had no adverse effects on the cells.

### 2.6. Stem Cell Differentiation into Embryoid Bodies

The capability of hESCs to differentiate into any cell within the body is essential for any application of regenerative medicine. To ensure that the MnEtP contrast agent caused no negative effects on the stem cells' ability to differentiate, labeled hESCs were differentiated into embryoid bodies. Confluent hESCs were labeled with 10 *μ*M MnEtP for 24 h or 40 *μ*M MnEtP for 30 min, washed three times with PBS, and transferred to 6-well untreated, uncoated tissue culture plates. The uncoated plates were then placed in the incubator on a shaker set at 60 rpm and left undisturbed for 5 days.

### 2.7. In Vivo Rat Study

All procedures were approved by our institutional animal care committee (protocol #41181) and were conducted in accordance with the Canadian Council on Animal Care. The in vitro studies showed that two different conditions provided excellent *T*_1_ reductions; therefore, both conditions were tested in vivo, namely, 10 *μ*M MnEtP for 24 h and 40 *μ*M MnEtP for 30 min. Cells were grown in T-75 flasks and were labeled at 70% confluency for either condition. Directly after labeling, the cells were washed three times, collected in a falcon tube, and centrifuged at 300 × *g* for 5 min. The supernatants were aspirated, and cells resuspended in 200 *μ*L of mTeSR™1 media, transferred to 1.5 mL Eppendorf tubes, placed on ice, and transported to the MR scanner. A female adult Sprague-Dawley rat (Charles River Laboratories) weighing 350 g was anesthetized on 3% isoflurane (Forene, Abbott Labs, Baar, Switzerland) in pure oxygen (2 L/min flow rate). Approximately 15 million labeled cells in 200 *μ*L mTeSR™1 were injected subcutaneously on the dorsal side below the shoulder blades. The unlabeled control cells were injected subcutaneously on the dorsal side posterior to the 10 *μ*M, 24 h labeled cell injection. The rat was placed prone in an 8-channel wrist coil, on a water blanket (HTP-1500, Adroit Medical Systems, London, TN) set at 41°C for maintenance of body temperature. A maintenance dose of 2% isoflurane in pure oxygen was used throughout imaging. Within one hour after cell injection, imaging was performed and completed. Forty 1 mm thick sagittal image slices were centered at midline. A 3D *T*_1_-weighted turbo field echo (TFE) sequence was acquired to detect the labeled cells: TR = 6.2 ms, TE = 3.3 ms, NSA = 16, FOV = 100 mm, and 0.6 × 0.6 mm in-plane resolution. To visualize the fluids within the injections, a 2D *T*_2_-weighted turbo spin-echo (TSE) sequence was acquired with the same pixel resolution and TR = 4000 ms, TE = 75 ms, and NSA = 2 with an echo train length = 16. Quantitative *T*_1_-mapping was performed using a variable flip-angle method [15].

### 2.8. Statistics

Differences in *T*_1_ and *T*_2_ relaxation times with labeling conditions were tested using one-way ANOVA with Tukey-Kramer post hoc analysis. A two-way ANOVA was used to determine significance for retention studies, with postlabeling interval and labeling condition as variables. Significance is reported at a *P* value of 5%.

## 3. Results

**Figure 1:**
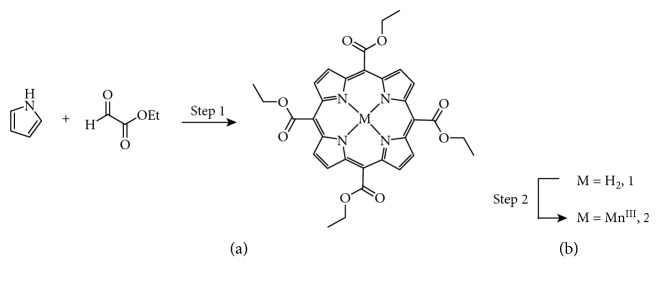
Schematic of synthesis of compound 2 (MnEtP). Reagents and conditions: step 1: (1) BF_3_OEt_2_, 1 h DCM 25°C; (2) DDQ, 2.5 h 10%; step 2: (1) MnCl_2_·4H_2_O, DMF, reflux 5 h; (2) 25°C, 16 h 85% [13, 16].

**Figure 2:**
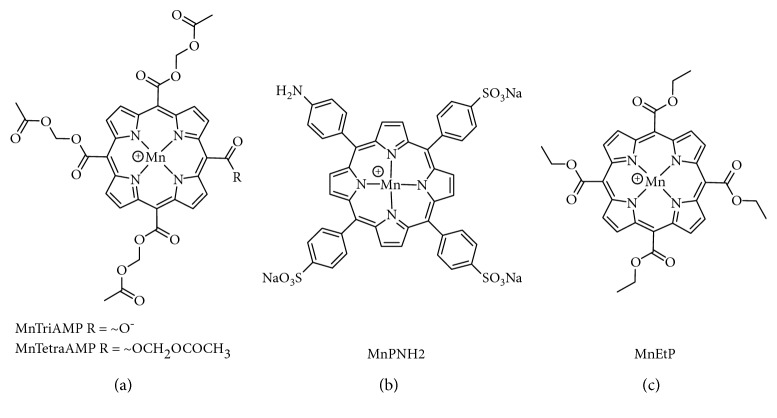
Chemical structures of contrast agents. (a) MnAMP is a 1 : 1 mixture of MnTriAMP and MnTetraAMP; (b) MnPNH2; (c) MnEtP.

Figure 1 shows the synthesis of compound 2, MnEtP [5, 10, 15, 20-tetrakis(ethoxycarbonyl)porphyrinato]manganese(III) chloride, done according to the literature [13, 16]. The first step is a condensation reaction between pyrrole and ethyl glyoxalate followed by in situ oxidation with DDQ to form the tetraethyl ester porphyrin, 1 in 10% yield. Manganese insertion was accomplished with 85% yield. The structure was confirmed by high resolution mass spectrometry (MS), and the purity was confirmed to be >95% by Mn flame atomic absorption spectroscopy and HPLC.  Figure 2 illustrates the chemical structure of MnEtP and those of previous cell-labeling agents, namely, MnTriAMP [5-carboxy-10, 15, 20-tris(acetoxymethylcarbonyl)porphyrinato]manganese(III) chloride, MnTetraAMP [5, 10, 15, 20-tetrakis(acetoxymethylcarbonyl)porphyrinato]manganese(III) chloride, and MnPNH2 [5-(4-aminophenyl)-10,15,20-tris(4-sulfonatophenyl)porphyrinato]manganese(III) chloride.

**Figure 3:**
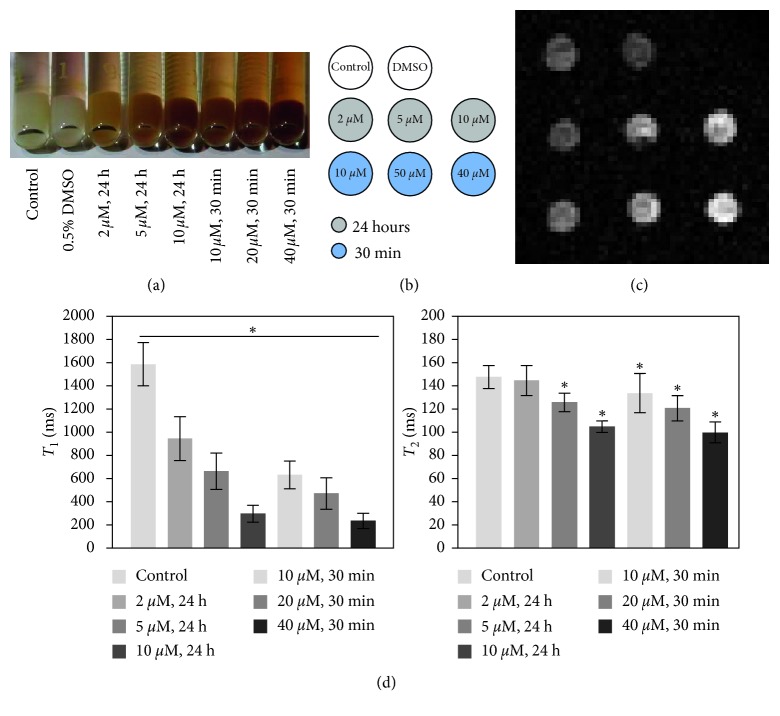
Optimization of conditions for labeling hESCs with MnEtP. (a) Photograph of labeled cell pellets prior to MRI; darker colour arises from MnEtP. (b) Legend for *T*_1_-weighted imaging. (c) *T*_1_-weighted spin-echo image of cell pellets (4 mm diameter) at 3 Tesla. (d) *T*_1_ and *T*_2_ relaxation times of labeled cells demonstrate efficient *T*_1_ reduction. Shown are mean values and standard deviations. Significant differences in *T*_1_ from control pellet to different labeling conditions are indicated (^*∗*^ *P* < 0.05).

Due to the hydrophobic nature of MnEtP, stock solutions of the agent were prepared in DMSO and infused into the media for cell labeling (concentration of DMSO in media = 0.5%). To control the effects of this solvent on cell labeling, control cells were cultured with 0.5% DMSO. As seen in Figure 3(a), both the unlabeled and DMSO labeled cell pellet were white in colour. In contrast, the pellets labeled for 24 h with 2 *μ*M, 5 *μ*M, and 10 *μ*M MnEtP show a gradual increase in red colour. Qualitatively, the darkest pellet with the most uptake resulted from the highest labeling concentration of 40 *μ*M, despite the short labeling interval of 30 min. The efficiency of MnEtP as a contrast agent is shown in Figures 3(b)–3(d). The trend seen with the differences in colour is confirmed by the MR results. Significant reduction in *T*_1_ relaxation times was achieved for all labeling conditions (*P* < 0.05), even with low labeling concentrations. Reductions in *T*_2_ were small, consistent with a *T*_1_ agent. The conditions with the largest *T*_1_ reductions, 10 *μ*M for 24 h and 40 *μ*M for 30 min, were chosen for the remainder of the study.

**Figure 4:**
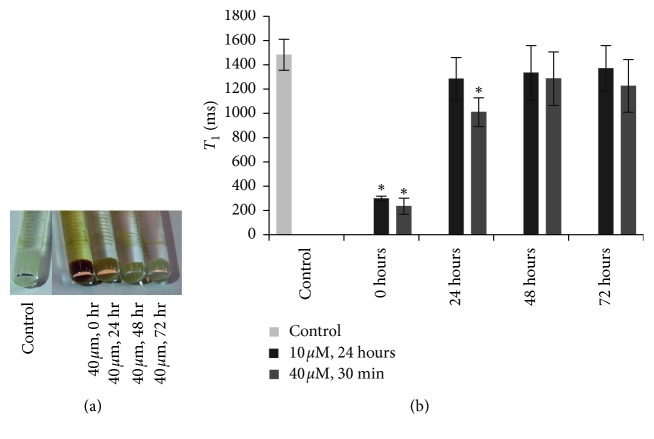
Retention of contrast agent in labeled hESCs. (a) Photograph of cell pellets labeled at 40 *μ*M for 30 min with MnEtP. (b) *T*_1_ relaxation times for unlabeled control cells and cells labeled at 10 *μ*M for 24 h and 40 *μ*M for 30 min, directly after labeling (0), and at 24, 48 and 72 h postlabeling. *T*_1_ reduction returned to baseline levels by 24 h for the 10 *μ*M labeling and by 48 h for the 40 *μ*M labeling conditions. Shown are mean values and standard deviations. Significant differences in *T*_1_ from control pellet are indicated (^*∗*^ *P* < 0.05).

A retention study of cells labeled at 10 *μ*M for 24 h showed that the *T*_1_ relaxation time returned to baseline levels 24 h postlabeling (Figure 4). In contrast, cells labeled at 40 *μ*M for 30 min maintained a substantial *T*_1_ reduction at 24 h and *T*_1_ only returned to the baseline by 48 h postlabeling. The cell pellets were quantified by ICP-AES (Table 1), and the results substantiate the *T*_1_ relaxation measurements.

To gain insight into the subcellular distribution of the agent, cells were fractionated into cytosolic, nuclear, and membrane components; the mass of Mn was then quantified.  Table 2 lists the relative percentage of Mn in the various fractions. For both labeling conditions, more than 90% of the agent was localized in the membrane fraction and less than 1% was in the nucleus.

**Figure 5:**
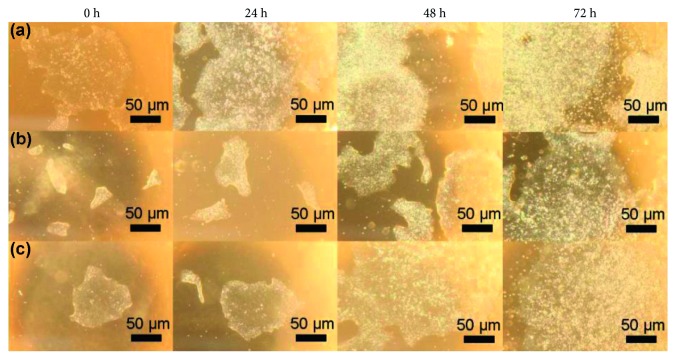
Phenotype of hESCs on light microscopy. Bright-field images of human embryonic stem cell colonies: (a) unlabeled control cells, (b) 10 *μ*M, 24 h, and (c) 40 *μ*M, 30 min directly after labeling and 24, 48, and 72 h postlabeling. Colony shape and cell morphology were unchanged after labeling (4x magnification).

Figure 5 illustrates bright-field images of representative colonies for labeled cells and unlabeled control taken immediately after labeling and at 1, 2, and 3 days postlabeling. The images show that labeling had no effect on colony shape, size, or distribution. Cell viability was also unaffected (Table 3).

**Figure 6:**
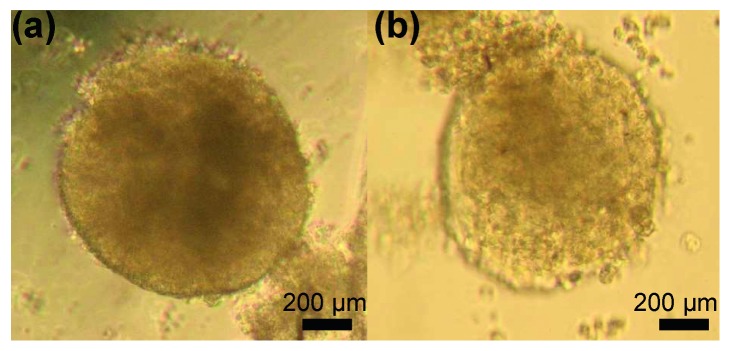
Differentiation capacity of MnEtP-labeled hESCs. Light microscopy of embryoid bodies 5 days postlabeling: (a) unlabeled control cells; (b) 10 *μ*M, 24 h MnEtP-labeled cells (4x magnification).

To verify that labeling had no detrimental effects on cell function, cells were differentiated into embryoid bodies. Both the unlabeled and labeled embryoid bodies appeared similar in size and shape, as seen on the bright-field images in Figure 6.

**Figure 7:**
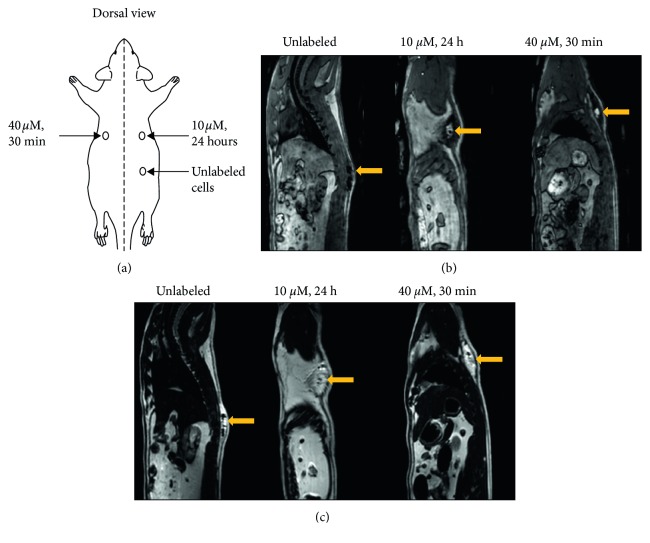
*In vivo* MR imaging of transplanted hESCs in an adult rat. (a) Location of subcutaneous injections of hESCs in 0.2 mL mTeSR1 media on the dorsal side of rat. (b) 3D *T*_1_-weighted TFE images without fat suppression clearly show contrast enhancement where the labeled cells were injected compared to unlabeled cells that were isointense against native tissue. (c) *T*_2_-weighted TSE images were acquired to identify fluid present in all injections. Yellow arrows indicate location of injected cells.

**Figure 8:**
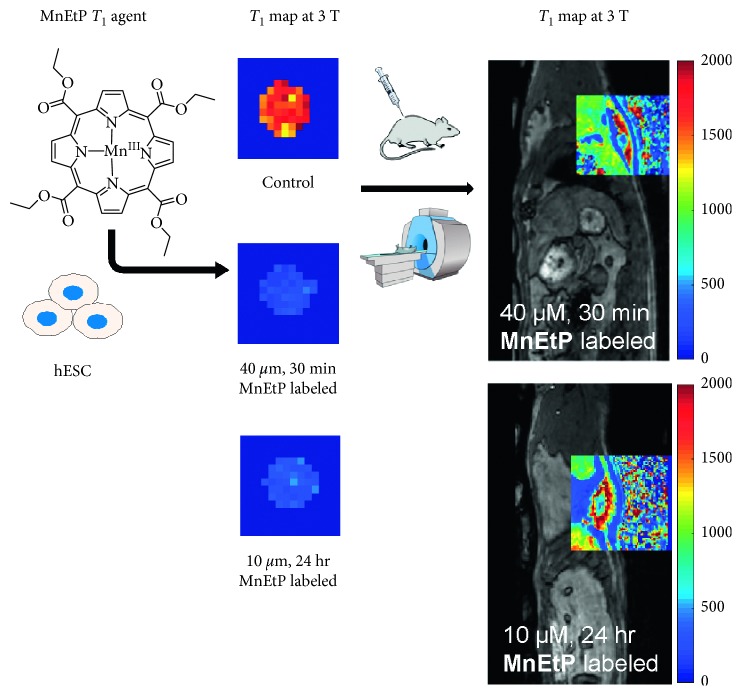
Comparison of reduction in *T*_1_ relaxation times in vitro and in vivo. An in vivo *T*_1_ map overlaid over the cell injection site shows similar reductions in *T*_1_ relaxation times (in units of ms) compared to cell pellet imaging. The same colour scale is used for both in vitro and in vivo maps.

*In vivo* MR imaging of a rat injected subcutaneously with labeled and unlabeled hESCs is shown in Figure 7. A schematic of injection locations (Figure 7(a)) is provided to facilitate interpreting the MR images. The labeled cells were clearly discerned on *T*_1_-weighted MRI as a hyperintense signal at the sight of injection, whereas unlabeled cells could not be detected (Figure 7(b)). *T*_2_-weighted images are also shown (Figure 7(c)) to illustrate that a *T*_2_ sequence is incapable of distinguishing the therapeutic cells as signal enhancement will arise not from the cells but from the fluid of the injectate.  Figure 8 shows an in vivo *T*_1_ map overlaid on an anatomical image of the rat and compares side-by-side the *T*_1_ reductions in a cell pellet. The injected stem cells had a *T*_1_ of 595 ± 138 ms and 488 ± 55 ms for the 10 *μ*M and 40 *μ*M conditions, respectively.

## 4. Discussion

Stem cells have been differentiated into a variety of cell types for treatment of complex and chronic conditions such as neurodegenerative diseases, autoimmune disease, and cancers [1, 2]. For example, pancreatic islet transplantation has shown success in treating type I diabetes [17]. However, progress in many cell-based therapies is stalled by several critical hurdles, foremost of which is lack of information on the fate of therapeutic cells once they are transplanted in the body. A large percentage of cells do not survive beyond the initial injection, and retention below 10% is common. Therefore, having a means to evaluate the initial success of cell therapy and where surviving cells are distributed is critical. The main objective for this work was to develop a positive-contrast *T*_1_ agent that provides efficient MR contrast, is biocompatible with both the cells and the organism receiving therapy, and can be synthesized in large quantities in labs focused on biomedical applications rather than synthesis of new agents—all the ingredients for a clinically translatable agent to guide cell therapy. To this end, we have developed a third-generation manganese porphyrin, MnEtP, with a scalable two-step synthesis, biocompatibility, efficient cell uptake, and unprecedented signal enhancement in vivo at very low labeling concentrations.

Our focus on *T*_1_ agents strives to achieve greater specificity and reliability of cell detection than is possible with SPIOs. However, due to an inherently lower sensitivity, *T*_1_ agents for cell tracking must be designed for high cell loading and efficient intracellular relaxivity. One agent developed by us, MnAMP, fulfills these criteria but was not amenable for larger animal studies due to difficulty in scaling up the synthesis. MnAMP was synthesized in 5 steps with an overall yield of only 4.7%. The new agent, MnEtP, was synthesized in 2 steps with almost double the yield at 8.5%. Structurally, MnEtP has four ethyl esters at the mesopositions of the porphyrin macrocycle, whereas MnAMP is a 1 : 1 mixture of 3 acetoxymethyl (AM) esters with one carboxylate and 4 AM esters at those positions. Therefore, MnEtP would be more hydrophobic since all four carboxylates are blocked by ethyl esters, and AM esters also have more oxygen atoms present per side chain. Furthermore, MnEtP has an overall positive charge due to the central manganese atom, which also promotes cell uptake via interactions with oligosaccharides on the cell surface. This new agent is also substantially more hydrophobic than MnPNH2, our second-generation agent, with three negatively charged sulfonates at the periphery. Therefore, MnPNH2 required relatively high labeling concentrations of 0.5 mM for 24 h compared to only 0.1 mM labeling for MnAMP for 24 h. The main advantage of MnPNH2 is its simple one-step synthesis from a commercially available apo form, 5-(4-aminophenyl)-10, 15, 20-tri(4-sulfonatophenyl)porphyrin, making this agent accessible to a wide range of research labs. As described above, MnEtP was synthesized in 2 steps and can also be made by researchers without extensive synthesis experience.

Amongst *T*_1_ cell-labeling agents, there have been a plethora of approaches based on both gadolinium (Gd) and manganese. In contrast to manganese porphyrins, which are naturally hydrophobic due to the large aromatic macrocycle, clinically approved Gd chelates are small, hydrophilic, and cell-impermeable. Several of these agents have been used for cell labeling but, due to their hydrophilicity, require transfection agents or electroporation with high incubation concentrations (5 mM–100 mM) and long labeling times for cell uptake [18]. To improve cell labeling efficiency, a variety of strategies have been used, including attachment of small chelates to lipophilic moieties [19, 20] and cell penetrating peptides [21, 22] to render them cell-permeable. However, these approaches use complex multistep syntheses, at least 5 steps, which severely limit the number of labs capable of making these agents [19, 21, 22]. An alternative strategy has been to incorporate large numbers of Gd-chelates into nanocarriers [23, 24] that have mechanisms for cell uptake. However, these are difficult to translate to clinical applications, since precise characterization of the constituents of these materials is difficult compared to well established characterization of small molecules. Aside from the complexity of synthesis, gadolinium toxicity is a major concern. Gd depositions have been found in brains of patients receiving repeated injections of linear clinical agents, raising concerns about the use of a toxic heavy metal [25, 26]. By comparison, porphyrins have evolved to chelate transition metals, specifically Fe^II^/Fe^III^, and since Mn^III^ is isoelectronic with Fe^II^, it is also thermodynamically and kinetically very stable [27].

The safety of MnEtP was confirmed from cell viability and differentiation assays. HPLC data from cell fractionation also confirmed that MnEtP was the only species detected (see Supplementary Materials available here): none of the apo-porphyrin was present, which points to the high stability of manganese porphyrins for cell labeling. In contrast, linear Gd-based contrast agents demetallate intracellularly, releasing the toxic Gd ion [28]. This risk is associated with nephrogenic systemic fibrosis [29, 30] in patients with reduced kidney function and has led to an increased demand for safer Gd-free alternatives. This is especially important for cell labeling where endosomal entrapment exposes the agent to much harsher conditions compared to the extracellular environment [28]. As a cell labeling agent, MnEtP demonstrated up to a six-fold reduction in *T*_1_ relaxation times compared to control cells. There was a clear trend of larger *T*_1_ reductions with higher labeling concentrations and/or longer labeling intervals. Even at very low labeling concentrations of 10 *μ*M (for 24 h) and 40 *μ*M (for 30 min), the *T*_1_ relaxation time dropped below 420 ms, which enabled high signal-to-noise imaging. Compared with previous agents, we now require at least ten times less material, a substantial advantage in terms of labeling large cell quantities. In fact, the material required for labeling an average therapeutic cell dose, ∼3.5 × 10^8^ cells for a 70 kg person [31], requires 69.2 mg MnPNH2, 10.6 mg MnAMP, and only 0.9 mg MnEtP for the 10 *μ*M, 24 h labeling condition. The two optimal conditions for MnEtP provide flexibility in terms of the labeling interval, depending on the requirements for specific applications.

The enhancement of labeled cells depends on the relaxivity of the agent, its intracellular concentration, and its subcellular localization. We, thus, determined both the Mn content and the subcellular distribution of MnEtP to elucidate the mechanisms of cellular contrast. Less than 1% of the agent accumulated in the nucleus, vital for maintaining stem cell function and viability. Subcellular localization experiments also confirmed that less than 5% of the agent resided in the cytosol and over 90% bound to the cell membrane. These results indicate that the high efficiency of *T*_1_ relaxation resulted predominantly from membrane-bound Mn exerting a decrease in water tumbling rate via fast water exchange. This mechanism of anchoring contrast agents to the cell membrane to enhance relaxation rate has also been exploited previously for Gd-based cell labeling agents [32, 33] to overcome quenching of relaxivity from intracellular compartmentalization [34].

An important consideration for all exogenous agents is the difficulty of tracking the status, function, and interaction of therapeutic cells with the host microenvironment at long intervals after cell therapy. Regardless of the presence or absence of mechanisms to retain the agent inside a cell, cell dilution effects inevitably precludes long-term monitoring. As each labeled cell divides, the concentration is approximately halved, and signal drops below the noise floor after several cell divisions. Monitoring over the longer term is feasible in only a few instances, such as tracking terminally differentiated, nondividing cells (e.g., T-effector cells for immunotherapy [35], pancreatic islets for diabetes [36]); here, a contrast agent designed with a cell retention mechanism, such as MnAMP, is valuable. However, most applications use proliferating cells [31] that do not benefit from a retention mechanism. This is demonstrated by MnAMP-labeled cancer cells where a reduction in cellular *T*_1_ relaxation was observed after 5 h in fresh media due to cell division [11]. For the large majority of cell-tracking applications, the most practical and tangible goal is to achieve sensitive and specific detection in the initial hours after transplantation to assess cell homing and distribution. In the case of MnEtP, the stem cells underwent cell division approximately once every 24 hours. Therefore, the loss of signal after 24 hours can be attributed to both the dilution effect and possibly the detachment of the agent from the cell membrane.

Future studies will focus on optimizing the labeling conditions for several different types of therapeutic cells, such as hematopoietic stem cells for nonischemic dilated cardiomyopathy [31]. These will be followed by in vivo studies to optimize the injection protocol for therapeutic cells in preclinical animal models of stem cell therapy.

In conclusion, we have reported on a new positive-contrast *T*_1_ agent, MnEtP, for specific and sensitive in vivo tracking of hESCs. The new agent enables highly efficient labeling of hESCs without negative effects on cell viability or differentiation. Sensitive detection of labeled cells was demonstrated in vivo with MR imaging of transplanted cells at 3 Tesla. Future studies will focus on labeling therapeutic cells for in vivo transplantation in preclinical animal models of stem cell therapy for regenerative medicine.

## Figures and Tables

**Table 1:** Quantification of intracellular Mn content by ICP-AES.

Incubation conditions	Retention period (h)	[Mn] (fg/cell)
Control	No labeling	BDL
10 *μ*M, 24 h	0	12.5
10 *μ*M, 24 h	24	2
10 *μ*M, 24 h	48	BDL
10 *μ*M, 24 h	72	BDL
40 *μ*M, 30 min	0	37
40 *μ*M, 30 min	24	7
40 *μ*M, 30 min	48	BDL
40 *μ*M, 30 min	72	BDL

Cell retention results up to 72 h postlabeling for optimized labeling conditions are shown (BDL: below detection limit).

**Table 2:** Subcellular distribution of MnEtP.

Fraction	Labeling condition	% Mn
Cytosol	Control	BDL
	10 *μ*M, 24 h	1.2
	40 *μ*M, 30 min	5.0

Nuclear	Control	BDL
	10 *μ*M, 24 h	0.6
	40 *μ*M, 30 min	0.6

Membrane	Control	BDL
	10 *μ*M, 24 h	98.2
	40 *μ*M, 30 min	94.5

Results shown are for two optimized labeling conditions. The mass of Mn/fraction is given as a percentage (BDL: below detection limit).

**Table 3:** Cell viability at 6 days postlabeling.

Labeling condition	% viability
Unlabeled control	82.1 ± 14.4
0.5% DMSO	82.0 ± 11.8
10 *μ*M, 24 h	90.5 ± 11.9
40 *μ*M, 30 min	89.9 ± 13.3

Viability was determined by trypan blue assay. Shown are mean values and standard deviations.

## Data Availability

The data used to support the findings of this study are available from the corresponding author upon request.

## Supplementary Materials

Supplementary Materials General methods, synthetic procedures, and original spectra for structural characterization; HPLC program for analysis of the cytosolic and nuclear cell fractions; and cell viability after MnEtP labeling.

## References

[1] Stoltz J. F., de Isla N., Li Y. P. (2015). Stem cells and regenerative medicine: myth or reality of the 21^st^ century. *Stem Cells International*.

[2] Behfar A., Crespo-Diaz R., Terzic A., Gersh B. J. (2014). Cell therapy for cardiac repair-lessons from clinical trials. *Nature Reviews Cardiology*.

[3] Angelos M. G., Kaufman D. S. (2015). Pluripotent stem cell applications for regenerative medicine. *Current Opinion in Organ Transplantation*.

[4] Murry C. E., Keller G. (2008). Differentiation of embryonic stem cells to clinically relevant populations: lessons from embryonic development. *Cell*.

[5] Srivastava A. K., Kadayakkara D. K., Bar-Shir A., Gilad A. A., McMahon M. T., Bulte J. W. M. (2015). Advances in using MRI probes and sensors for in vivo cell tracking as applied to regenerative medicine. *Disease Models & Mechanisms*.

[6] Goodfellow F. T., Simchick G. A., Mortensen L. J., Stice S. L., Zhao Q. (2016). Tracking and quantification of magnetically labeled stem cells using magnetic resonance imaging. *Advanced Functional Materials*.

[7] Ma N., Cheng H., Lu M. (2015). Magnetic resonance imaging with superparamagnetic iron oxide fails to track the long-term fate of mesenchymal stem cells transplanted into heart. *Scientific Reports*.

[8] Muja N., Bulte J. W. M. (2009). Magnetic resonance imaging of cells in experimental disease models. *Progress in Nuclear Magnetic Resonance Spectroscopy*.

[9] Giesel F. L., von Tengg-Kobligk H., Wilkinson I. D. (2006). Influence of human serum albumin on longitudinal and transverse relaxation rates (R1 and R2) of magnetic resonance contrast agents. *Investigative Radiology*.

[10] Loai S., Haedicke I., Mirzaei Z., Simmons C. A., Zhang X.-A., Cheng H. L. (2016). Positive-contrast cellular MRI of embryonic stem cells for tissue regeneration using a highly efficient *T*
_1_ MRI contrast agent. *Journal of Magnetic Resonance Imaging*.

[11] Haedicke I. E., Li T., Zhu Y. L. K. (2016). An enzyme-activatable and cell-permeableMnIII-porphyrin as a highly efficient *T*
_1_ MRI contrast agent for cell labeling. *Chemical Science*.

[12] Venter A., Szulc D. A., Loai S., Ganesh T., Haedicke I. E., Cheng H.-L. M. (2018). A manganese porphyrin-based*T*
_1_ contrast agent for cellular MR imaging of human embryonic stem cells. *Scientific Reports*.

[13] Trova M. P., Gauuan P. J. F., Pechulis A. D. (2003). Superoxide dismutase mimetics—part 2: synthesis and structure-activity relationship of glyoxylate- and glyoxamide-derived metalloporphyrins. *Bioorganic & Medicinal Chemistry*.

[14] Choudhary S., Keshavarzian A., Yong S. (2001). Novel antioxidants zolimid and AEOL11201 ameliorate colitis in rats. *Digestive Diseases and Sciences*.

[15] Cheng H.-L. M., Wright G. A. (2006). Rapid high-resolution*T*
_1_ mapping by variable flip angles: accurate and precise measurements in the presence of radiofrequency field inhomogeneity. *Magnetic Resonance in Medicine*.

[16] Cheng W., Haedicke I. E., Nofiele J. (2014). Complementary strategies for developing Gd-freehigh-field *T*
_1_ MRI contrast agents based on MnIII porphyrins. *Journal of Medicinal Chemistry*.

[17] Shapiro A. M. J., Lakey J. R. T., Ryan E. A. (2000). Islet transplantation in seven patients with type 1 diabetes mellitus using a glucocorticoid-free immunosuppressive regimen. *New England Journal of Medicine*.

[18] Crich S. G., Biancone L., Cantaluppi V. (2004). Improved route for the visualization of stem cells labeled with a Gd-/Eu-Chelate as dual (MRI and fluorescence) agent. *Magnetic Resonance in Medicine*.

[19] Joshi R., Mishra R., Pohmann R., Engelmann J. (2010). MR contrast agent composed of cholesterol and peptide nucleic acids: design, synthesis and cellular uptake. *Bioorganic & Medicinal Chemistry Letters*.

[20] Yamane T., Hanaoka K., Muramatsu Y. (2011). Method for enhancing cell penetration of Gd^3+^-based MRI contrast agents by conjugation with hydrophobic fluorescent dyes. *Bioconjugate Chemistry*.

[21] Endres P. J., MacRenaris K. W., Vogt S., Meade T. J. (2008). Cell-permeable MR contrast agents with increased intracellular retention. *Bioconjugate Chemistry*.

[22] Mishra R., Su W., Pohmann R. (2009). Cell-penetrating peptides and peptide nucleic acid-coupled MRI contrast agents: evaluation of cellular delivery and target binding. *Bioconjugate Chemistry*.

[23] Figueiredo S., Cutrin J. C., Rizzitelli S. (2013). MRI tracking of macrophages labeled with glucan particles entrapping a water insoluble paramagnetic Gd-based agent. *Molecular Imaging and Biology*.

[24] Aime S., Castelli D. D., Crich S. G., Gianolio E., Terreno E. (2009). Pushing the sensitivity envelope of lanthanide-based magnetic resonance imaging (MRI) contrast agents for molecular imaging applications. *Accounts of Chemical Research*.

[25] Frenzel T., Apte C., Jost G., Schöckel L., Lohrke J., Pietsch H. (2017). Quantification and assessment of the chemical form of residual gadolinium in the brain after repeated administration of gadolinium-based contrast agents. *Investigative Radiology*.

[26] Vergauwen E., Vanbinst A. M., Brussaard C. (2018). Central nervous system gadolinium accumulation in patients undergoing periodical contrast MRI screening for hereditary tumor syndromes. *Hereditary Cancer in Clinical Practice*.

[27] Berezin M. B. (2001). Thermochemistry of solution of Fe(III) and Mn(III) complexes with natural porphyrins. *Russian Journal of General Chemistry*.

[28] Di Gregorio E., Gianolio E., Stefania R., Barutello G., Digilio G., Aime S. (2013). On the fate of MRI Gd-based contrast agents in cells: evidence for extensive degradation of linear complexes upon endosomal internalization. *Analytical Chemistry*.

[29] Daftari Besheli L., Aran S., Shaqdan K., Kay J., Abujudeh H. (2014). Current status of nephrogenic systemic fibrosis. *Clinical Radiology*.

[30] Garcia J., Liu S. Z., Louie A. Y. (2017). Biological effects of MRI contrast agents: gadolinium retention, potential mechanisms and a role for phosphorus. *Philosophical Transactions Series A, Mathematical, Physical, and Engineering Sciences*.

[31] Naumova A. V., Modo M., Moore A., Murry C. E., Frank J. A. (2014). Clinical imaging in regenerative medicine. *Nature Biotechnology*.

[32] Carney C. E., MacRenaris K. W., Meade T. J. (2015). Water-soluble lipophilic MR contrast agents for cell membrane labeling. *JBIC Journal of Biological Inorganic Chemistry*.

[33] Carney C. E., MacRenaris K. W., Mastarone D. J., Kasjanski D. R., Hung A. H., Meade T. J. (2014). Cell labeling via membrane-anchored lipophilic MR contrast agents. *Bioconjugate Chemistry*.

[34] Kok M. B., Hak S., Mulder W. J. M., van der Schaft D. W. J., Strijkers G. J., Nicolay K. (2009). Cellular compartmentalization of internalized paramagnetic liposomes strongly influences both *T*
_1_ and *T*
_2_ relaxivity. *Magnetic Resonance in Medicine*.

[35] Gattinoni L., Speiser D. E., Lichterfeld M., Bonini C. (2017). T memory stem cells in health and disease. *Nature Medicine*.

[36] Shapiro A. M. J., Pokrywczynska M., Ricordi C. (2016). Clinical pancreatic islet transplantation. *Nature Reviews Endocrinology*.

