# Supplementary material for: An Efficient T 1 Contrast Agent for Labeling and Tracking Human Embryonic Stem Cells on MRI
Source: Contrast Media Mol Imaging. 2019 Jun 16;2019:3475786. doi: 10.1155/2019/3475786 (PMC6604499; doi:10.1155/2019/3475786)
Supplement: Supplementary Materials — General methods, synthetic procedures, and original spectra for structural characterization; HPLC program for analysis of the cytosolic and nuclear cell fractions; and cell viability after MnEtP labeling. [file 3475786.f1.docx]

**Supporting Information**

**An Efficient *T*_1_ Contrast Agent for Labeling and Tracking Human Embryonic Stem Cells on MRI**

**Table of Contents**

[General Methods S2](#_Toc535849146)

[Synthesis of [5,10,15,20-tetrakis(ethoxycarbonyl)porphyrinato]manganese(III) chloride, **2**, **MnEtP**. S2](#_Toc535849147)-S3

[High Performance Liquid Chromatography S3](#_Toc535849148)

[Quantification of Intracellular Manganese Content by inductively coupled plasma-atomic emission spectroscopy (ICP-AES) S4](#_Toc535849149)

[Analysis of Cell Fractions by HPLC S4](#_Toc535849150)-S6

[Cell Viability and Proliferation S6](#_Toc535849151)

[Structural Characterization S7](#_Toc535849152)-S8

[References S9](#_Toc535849153)

# General Methods

All reagents and solvents used for synthesis were of reagent grade or better and were used without further purification unless stated otherwise. Ethyl glyoxalate (50% in toluene) and triethylamine were purchased from Acros Organics. Pyrrole, boron trifluoride diethyl etherate and manganese (II) chloride tetrahydrate and deuterated chloroform were purchased from Sigma-Aldrich. 2,3-dichloro-5,6-dicyano-1,4-benzoquinone (DDQ) was purchased from Alfa Aesar. Sterile dimethyl sulfoxide (DMSO) was purchased from Bioshop (Cat. No. DMS666.100). Dulbecco’s phosphate buffered saline (without Ca, Mg, sterile filtered and endotoxin tested) was purchased from VWR (Cat. No. 02-0119-1000). All solutions were prepared with water from a MilliQ system (18.2 MΩ cm). Ammonium acetate and high-performance liquid chromatography (HPLC) grade acetonitrile were purchased from Caledon Labs. HPLC grade methanol was purchased from Fisher Scientific. Thin layer chromatography was carried out on pre-coated aluminium plates of Silica Gel 60 F254 from Merck. Data for structural characterizations were obtained using the research facilities at University of Toronto-St George (Department of Chemistry) and UT-Scarborough (Traces Center). NMR spectra were recorded on a Brucker-500 MHz spectrometer. UV spectra were recorded on a Beckman Coulter DU 800 Spectrophotometer. High resolution mass spectra were obtained from an Agilent 6538 Q-TOF system. Flame atomic absorption spectroscopy (FAAS) was carried out on a PerkinElmer AAnalyst 100 system. Analytical reverse phase (RP) HPLC was done on an Agilent 1100 system equipped with an Agilent 1100 series diode array UV/Vis detector and an Eclipse C-18 reverse phase column (4.6 mm x 150 mm, 5 µm).

# Synthesis of [5,10,15,20-tetrakis(ethoxycarbonyl)porphyrinato]manganese(III) chloride, **2**, **MnEtP**.

The final product was synthesized, as we previously reported,^1, 2^ in two steps from a modified literature method. ^3^ Ethyl glyoxalate (50% in toluene, 1.88 mL, 9.4 mmol) and freshly distilled pyrrole (0.65 mL, 9.4 mmol) were stirred at room temperature in the dark. After 10 min, boron trifluoride diethyl etherate (0.4 mL, 3.1 mmol) was added drop-wise. The progress of the reaction was monitored by UV spectroscopy. After 1.25 hours (h), DDQ (1.60 g, 7.05 mmol) was added and the reaction was left stirring for 2.25 h. The reaction was quenched with triethyl amine (0.43 mL, 3.06 mmol) and concentrated by rotary evaporation. The crude solution was filtered over celite and basic alumina using DCM as the eluent. Purification by column chromatography on silica gel with DCM as eluent gave 141 mg (10% yield) of **1** as a black-purple solid. NMR (**Figure S8**) and UV spectra matched literature values.

Compound **1** was dissolved in 2 mL DMF. MnCl_2_^●^4H_2_O (18 mg, 89.2 µmol) was added and the reaction refluxed open to air for 5 h. The reaction was stirred at 25 ^o^C for an additional 16 h to allow for complete oxidation. Distillation of DMF under reduced pressure and purification with stepwise gradient column chromatography (eluting with 100% DCM – 7% MeOH/DCM) on silica gel gave 17 mg (85%) of **2**, **MnEtP**, as a black-brown solid. Characterization by UV and high-resolution mass spectrometry (HRMS) confirmed the product (**Figure S9-S10**) and matched the literature.^1^ The purity of the final product was analyzed and determined to be ≥95% pure using Mn-FAAS and RP-HPLC (**Figure S1**).

# High Performance Liquid Chromatography

The purity of the final product was analyzed and determined to be ≥ 95% pure using analytical HPLC with a flow rate of 0.8 mL/min. The product was detected at 452 nm and ran through at a flow rate of 0.8 mL/min, with MeOH as the organic phase and 10 mM NH_4_Ac (pH 6.8) as the aqueous phase, for solvent gradients see **Table S1**. Due to limited solubility **MnEtP** was dissolved in MeOH and infused into ultrapure water.

| Time (min) | 0 | 3 | 10 | 18 |
| --- | --- | --- | --- | --- |
| MeOH:H_2_O | 5:95 | 5:95 | 80:20 | 100:0 |

**Table S1.** Solvent gradients used for HPLC.


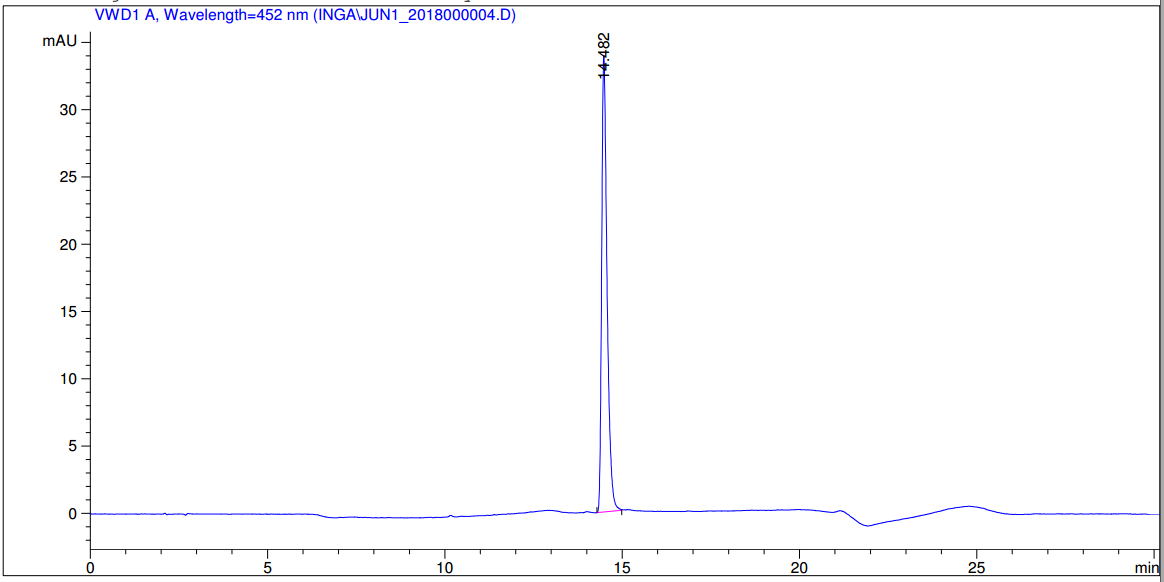


**Figure S1.** HPLC chromatogram of **MnEtP**, **2**. Elution occurred at 14.5 min with 100% purity.

# Quantification of Intracellular Manganese Content by inductively coupled plasma-atomic emission spectroscopy (ICP-AES)

After MRI, the cell pellets were centrifuged at 300 x*g* for 5 min. The supernatant was removed, and 600 µL nitric acid TraceSELECT (Fluka Analytical) was added. The cells were digested at 40^o^C, with sonication, for 7 h until the mixture became clear. The solutions were diluted to 2% w/v nitric acid (HNO_3_), filtered over 0.22 µm polyether sulfone (PES) syringe filter and analyzed on an ICP atomic emission spectrometer (Optima 7300 DV ICP AES). A 1000 mg/mL manganese standard (2% w/v HNO_3_) Titrisol (Aldrich) was used for preparation of standard working solutions. Nitric acid was used for stabilization of samples and working standards. Standard solutions for calibration purposes were prepared by proper dilution with 2% w/v HNO_3_ solution. A rinsing step was included prior to withdrawal of each aliquot. Measurements were performed in triplicate. The cellular elemental concentration was determined by dividing the total content by the number of cells. The endogenous Mn content was below the limit of detection (10 ppb).

# Analysis of Cell Fractions by HPLC

The HPLC method, used for analysis of the cytosolic and nuclear fractions, is in table S1. The product was detected at 452 nm and ran through at a flow rate of 0.8 mL/min, with MeOH as the organic phase and 10 mM NH_4_Ac (pH 6.8) as the aqueous phase.


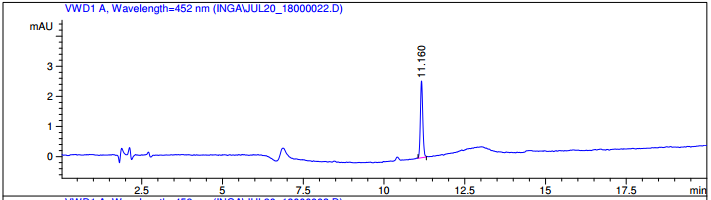


**Figure S2.** HPLC chromatogram of the cytosolic fraction from the unlabeled control cells, background peak at 11.2 min.


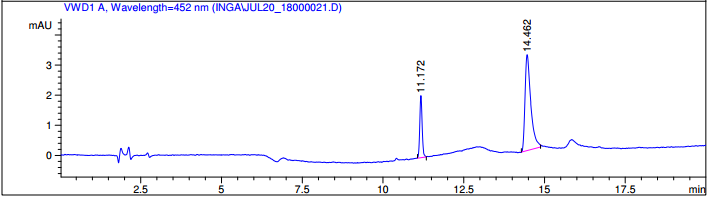


**Figure S3.** HPLC chromatogram of the cytosolic fraction from **MnEtP**, 10 µM, 24 hours, labeled cells, background peak at 11.2 min, **MnEtP** at 14.5 min.


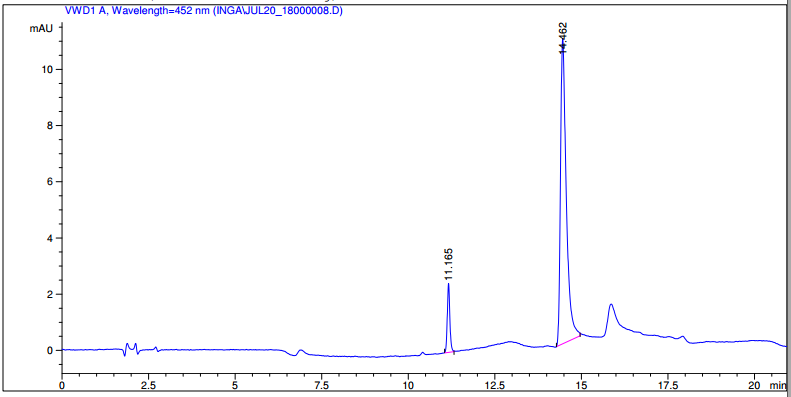


**Figure S4.** HPLC chromatogram of the cytosolic fraction from **MnEtP**, 40 µM, 30 min, labeled cells, background peak at 11.2 min, **MnEtP** at 14.5 min.


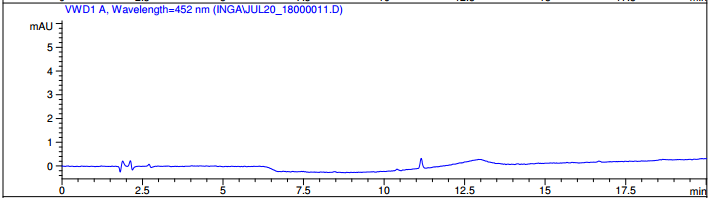


**Figure S5.** HPLC chromatogram of the nuclear fraction from the unlabeled control cells, background peak at 11.2 min.


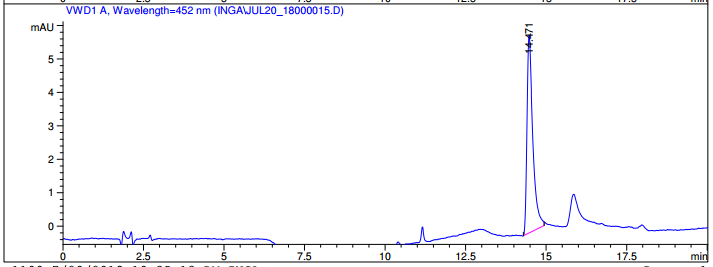


**Figure S6.** HPLC chromatogram of the nuclear fraction from **MnEtP**, 10 µM, 24 hours, labeled cells with a background peak at 11.2 min.


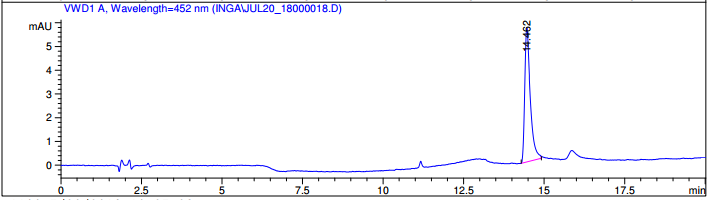


**Figure S7.** HPLC chromatogram of the nuclear fraction from **MnEtP**, 40 µM, 30 min, labeled cells with a background peak at 11.2 min.

# Cell Viability and Proliferation

Cell viability and proliferation were evaluated on **MnEtP-**labeled hESCs 6 days post-labeling to ensure no detrimental effects from the agent. Labeling was performed at ~30% confluency to allow adequate space for continued growth. At day 6 post-labeling, the confluency was ~90% and the cells were washed, detached, collected and centrifuged at 300 x*g* for 5 min. The media was aspirated, the cells were suspended with 6 mL fresh media, placed on ice and brought to the cell counter. The cells were resuspended and 50 µL of the suspension was diluted to 500 µL with D-PBS for cell counting with an automated cell counter (Vi-CELL R 2.03- Beckman Coulter). Fifty counts were taken for each sample, thereby generating results for the total cell number, viable cell count, nonviable cell count, and concentration of cells/mL. An average viability count was reported. The counter was set so that cells with a diameter of 5-50 µm were detected. The field of view was set to 831 x 611 µm, and 1280 x 960 images were generated.

# Structural Characterization


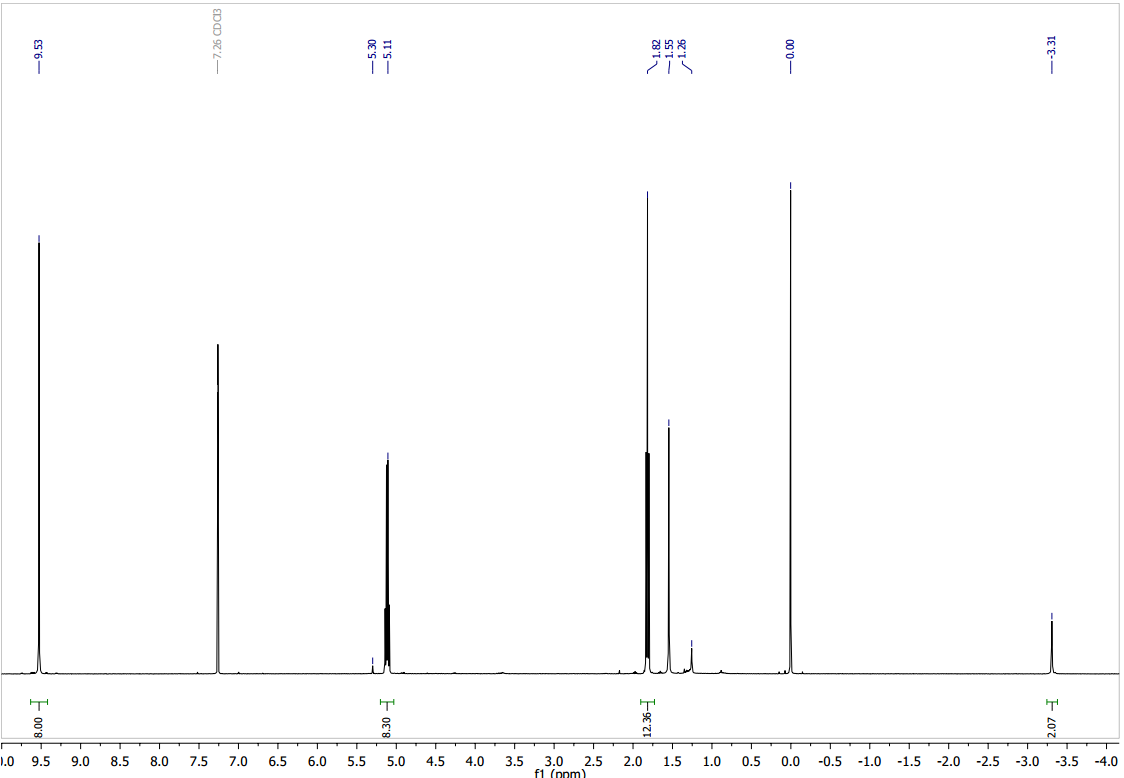


**Figure S8.** ^1^H NMR spectrum of **1** in CDCl_3_. The residual solvent peaks are chloroform (7.26 ppm), dichloromethane (5.30 ppm), water (1.56ppm) and hexanes (1.26 ppm).


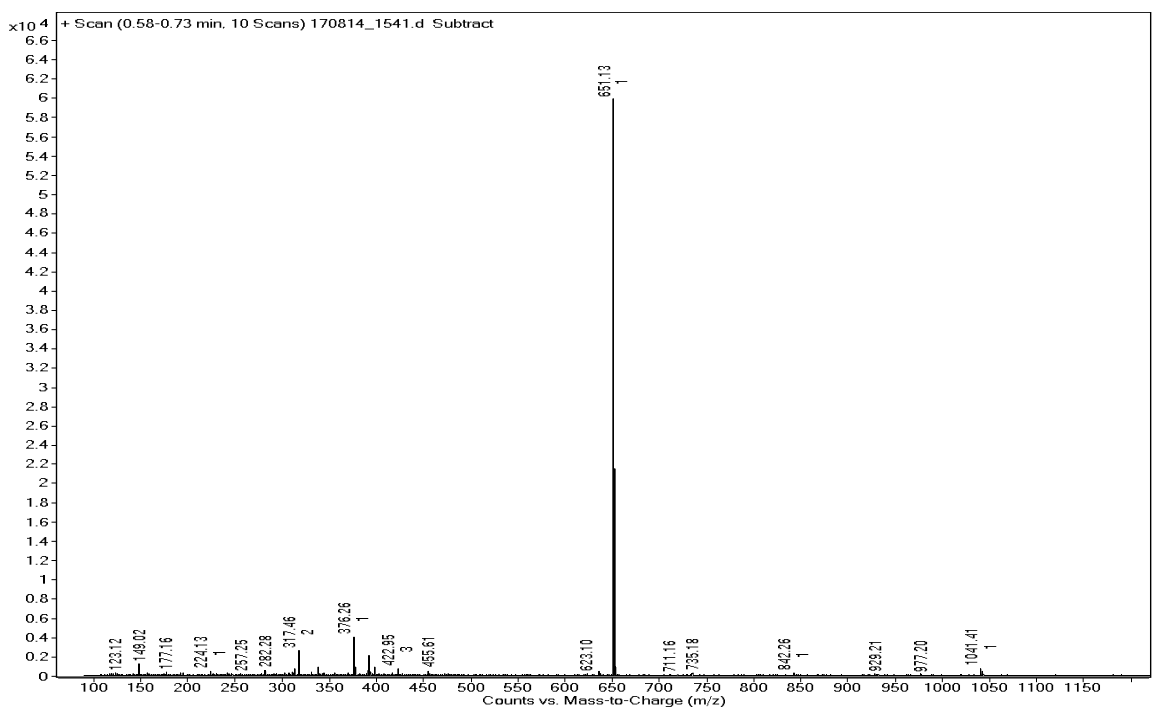


**Figure S9.** ESI-MS positive mode m/z = 651.13, [M^+^] for **MnEtP**, **2**.


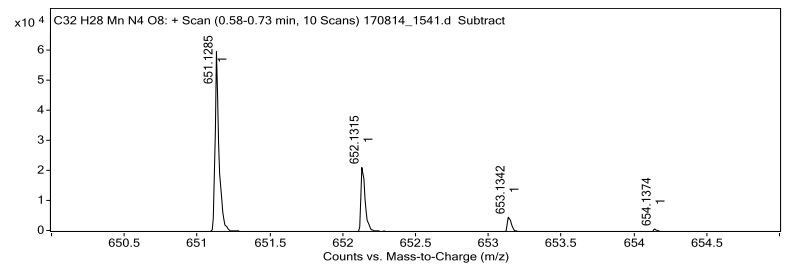


**Figure S10.** HR ESI-MS Positive mode m/z = 651.1285, [M^+^] for **MnEtP**, **2**.

# References

(1) Cheng, W.; Haedicke, I. E.; Nofiele, J.; Martinez, F.; Beera, K.; Scholl, T. J.; Cheng, H. L.; Zhang, X. A. (2014) Complementary strategies for developing Gd-free high-field T(1) MRI contrast agents based on Mn(III) porphyrins. *J. Med. Chem. 57* (2), 516-20.

(2) Haedicke, I. E.; Li, T.; Zhu, Y. L. K.; Martinez, F.; Hamilton, A. M.; Murrell, D. H.; Nofiele, J. T.; Cheng, H.-L. M.; Scholl, T. J.; Foster, P. J. et al. (2016) An enzyme-activatable and cell-permeable MnIII-porphyrin as a highly efficient T1 MRI contrast agent for cell labeling. *Chemical Science 7* (7), 4308-4317.

(3) Trova, M. P.; Gauuan, P. J. F.; Pechulis, A. D.; Bubb, S. M.; Bocckino, S. B.; Crapo, J. D.; Day, B. J. (2003) Superoxide dismutase mimetics. Part 2: synthesis and structure-activity relationship of glyoxylate- and glyoxamide-derived metalloporphyrins. *Bioorg. Med. Chem.* 11 (13), 2695-2707.
